# Contextual image caption creation using object positional embedding and generative models

**DOI:** 10.1371/journal.pone.0353466

**Published:** 2026-07-09

**Authors:** Muhammad Danyal, Muhammad Roman, Abdul Shahid, Muhammad Yahya

**Affiliations:** 1 Institute of Computing, Kohat University of Science and Technology, Kohat, Pakistan; 2 Bristol Research and Innovation Laboratory (BRIL), Toshiba Europe Ltd., Bristol, England; 3 School of Business, South East Technological University (SETU), Waterford, Ireland; 4 Data Science Institute, University of Galway, Galway, Ireland; King Abdulaziz University Faculty of Engineering, SAUDI ARABIA

## Abstract

Automated image captioning remains a challenge, as it enables machines to generate context-aware textual descriptions of visual content. Traditional deep learning approaches often rely on lexical overlap and fail to capture semantic relationships among objects, leading to captions that lack contextual richness. This study proposes an encoder–decoder framework that integrates YOLOv5 with a generative transformer to generate descriptive image captions. The proposed model was evaluated against two baselines: CNN-LSTM (M1) and a BERT-based transformer model (M2). M1 achieves BLEU-1 (0.45) and ROUGE-L (0.42) but demonstrates limited semantic understanding with METEOR (0.18) and SPICE (0.07). M2 improves with higher METEOR (0.24) and CIDEr (0.62), although its BLEU scores remain low. The proposed model achieves the highest CIDEr (1.10) and SPICE (0.25), reflecting superior semantic understanding and better capture of object relationships. Despite a lower BLEU (0.40), it significantly outperforms traditional methods in caption quality. To further validate these results, we conducted an expert-based evaluation to assess semantic accuracy, visual grounding, and caption usefulness. The proposed model achieved 93% accuracy in expert evaluations across 500 images, indicating strong contextual alignment with human interpretation. Additionally, we employed exploratory data analysis to examine and visualize the text captions, aiming to gain a deeper understanding of the optimal caption.

## Introduction

In the age of digital media, a myriad of images from online platforms, news outlets, and advertisements capture the public’s attention. These visual canvases present a challenge, as viewers must independently discern and comprehend their inherent narratives. Interestingly, the majority of these photographs lack any accompanying descriptions. However, despite the absence of detailed captions, humans can grasp the meaning and essence of these images. This innate capacity to comprehend visual content without explicit textual guidance demonstrates our perceptual and cognitive capabilities.

Our brains are highly adept at processing and making sense of visual information, allowing us to derive meaning, infer context, and extract relevant insights from the images. Nonetheless, there are situations where individuals may desire automated image captions generated by machines. This could be particularly useful for accessibility purposes or when individuals prefer a more structured approach to understanding visual content. In such cases, the machine responsible for generating these captions must be able to comprehend and interpret some form of the caption, whether it is derived from the content surrounding the image or based on other contextual cues, and to understand and generate captions.

Machines can bridge the gap between textual representation and visual information, offering a valuable tool for those seeking automated assistance in interpreting images. Image captioning creates natural language descriptions of the main elements, their characteristics, and the interrelationships of the elements in a picture or scene using the information contained within. A good caption provides the underlying semantic meanings [1], identifies the objects in an image, and describes their actions and the effects on the surrounding objects.

Image Interpretation involves extracting meaningful data from visual information using techniques like object identification, pattern recognition, and inference. Object detection or identification in computer vision consists of locating and classifying objects in images or video frames using bounding boxes, thereby providing precise localization. Two primary methods, regional proposals and regression/classification, enable diverse approaches to object detection and description within images.

The region proposal-based framework follows a two-step process similar to the human brain’s attention mechanism. It first scans the entire image, focusing on areas of interest, akin to human attention to essential details. Then, using a sliding-window approach, a CNN is applied to predict bounding-box coordinates directly. Some of the models in this category are discussed here. Region-based Convolutional Neural Network (R-CNN) improves bounding box accuracy and uses deep neural networks to extract meaningful features [2]. Some use selective search to extract regions of interest, which are then merged iteratively.

The Spatial Pyramid Pooling Network (SPP-Net) employs spatial pyramid pooling to improve object recognition accuracy by partitioning images and aggregating features across sub-regions [3]. Fast R-CNN offers a unified architecture that extracts region proposals from a convolutional feature map and passes them through RoI pooling [2]. It predicts region class and bounding box offset values using a softmax layer, processing data faster than R-CNN. Faster R-CNN integrates a region proposal network (RPN) into the architecture, directly generating region recommendations within the network for more effective and precise object detection [4]. Region-based Fully Convolutional Network (R-FCN) employs a position-sensitive score map for object detection, effectively leveraging computational resources while achieving high accuracy [5].

The Feature Pyramid Network (FPN) builds a pyramid of feature maps at multiple scales, enabling efficient management of objects of varying sizes by incorporating a hierarchical structure and facilitating information flow across different levels of the network [6]. Mask R-CNN is utilised for segmentation and object detection, adding an extra level of prediction to generate pixel-level masks for accurate instance segmentation within an image [7]. It can handle tasks like human pose estimation with minor modifications.

For real-time object recognition, one-stage detectors offer a clear advantage over traditional region-proposal methods because they directly predict object classes and bounding boxes in a single step. Frameworks such as You Only Look Once (YOLO) [8] and Single Shot MultiBox Detector (SSD) [9] efficiently map image pixels to detection outputs without requiring multi-stage processing, making them suitable for applications like surveillance and autonomous systems. In practice, researchers often select the YOLO variant that best balances speed and accuracy for their task rather than focusing on historical differences between versions. SSD similarly provides fast detection by using a single deep network with multi-scale feature maps. Thus, in our proposed pipeline, we used Yolov5 [10] for effective feature extraction.

The language-generation aspect of image captioning will be examined using conventional machine learning and deep learning approaches. This section provides an overview of two methods from the literature: retrieval-based and language-template-based techniques. Retrieval-based techniques match the query image against images in the training set and identify potential descriptions linked to similar photos. After manual titling and captioning of downloaded images, the final product is generated from the most comparable pictures and captions in the network collection [8].

In image captioning, a method employs a two-step process: sorting images by labels or attributes to create visually related clusters, followed by matching each image to suitable captions for meaningful descriptions. This approach ensures that captions for the sorted images are relevant and contextually aligned [9]. They employed visual similarity and word density to find matching image descriptions within the dataset. The highest-rated caption, based on word density, determines the selected image description, yet this method assumes captions align perfectly with the image representation, which is often inaccurate [10].

Various methods aim to improve caption sentences by parsing them into descriptive phrases and selecting relevant phrases from descriptions of similar images using a trained model [11]. Finally, relevant phrases are combined to generate a new caption sentence using a tree structure to locate pertinent words gathered from previous picture captions, which are carefully combined to create fresh sentences [12]. Retrieval-based techniques rely on existing datasets and struggle to generate new phrases, often struggling to find similar images within them.

Language Template-Based methods analyse visual features such as objects, relationships, and attributes, and then generate captions using a language model. Multiple candidate sentences are generated and ranked based on their descriptive quality, with emphasis on extracting key visual features and generating captions. Using a language model, techniques accurately depict an image’s content by first detecting object locations, classifying them based on visual attributes, and generating a natural-language description via conditional random field (CRF) prediction [13].

Image analysis was previously performed using manually engineered feature extraction techniques and rule-based caption generation methods such as decision trees. Object nouns are categorized to identify descriptive content, organised based on attributes, and detailed sentences are generated using a trigram language model [14]. The method employs a multimodal similarity detector trained on image caption datasets, leveraging visual, linguistic, and multimodal cues. It utilises explanations derived from multiple learning instances, based on images and associated descriptions, integrating them into a language model to reorder and select the most relevant descriptions, thereby enhancing quality and relevance through multimodal cues [15].

Early image captioning studies used search-based and language template-based methods, each with limitations. Recent interest in neural networks stems from deep learning’s remarkable progress in various fields, offering a departure from traditional approaches. Exploring different deep-learning strategies for automatic picture captioning requires substantial computational and structural resources to identify practical, effective solutions.

Certain studies strive to construct holistic models integrating high-level semantic representations of query images. Their associated natural language counterparts, with a post-CNN component emphasising rich semantic features rather than direct image captioning, supplemented by the identification of the top 256 frequently used words in training set descriptions and the integration of a detector LSTM for enhanced accuracy and semantic information extraction [16].

The TCIC system facilitates communication of high-level cross-modality semantics via Theme Nodes (TTN) memory vectors for image captioning, integrating visual scene graph data and textual concepts for representation learning and caption generation using a shared transformer-based decoder [17,18]. To explore contextual interactions among various visual relationships between objects using semantic relation graphs, focusing on understanding comprehensive interactions and employing region-based bidirectional encoder BERT transformers to represent object interactions without additional relational annotations [19,20].

Attention-based approaches mimic human visual attention, focusing on key aspects of images to improve caption generation. By integrating attention mechanisms, models prioritize crucial visual elements, resulting in contextually relevant descriptions. This method ensures captions are informed by the most salient parts of the input picture, enhancing the accuracy and meaningfulness of the generated output. The framework uses a CNN and LSTM to generate image descriptions by first encoding images with GoogleNet [21], then decoding them with an LSTM, selecting high-frequency words from the output, and generating descriptions [22].

Integrating dual attention processes, combining visual and textual attention, enhances the model’s ability to prioritise critical textual details while selectively focusing on relevant visual elements. This integration improves consistency, cohesion, and comprehension, resulting in more comprehensive and accurate representations of both textual and visual content [23]. A special method for captioning images that are based on crucial visual information. It combines low-level elements, such as picture quality (sharpness and colour), with high-level ones, such as motion classification and face recognition, to determine image focus. Current methods for photograph captioning focus solely on linguistic rules rather than on visuals, leaving out the image’s context [24].

Transformer-based models have become the standard approach for sequence processing, particularly in applications such as machine translation and natural language understanding. Recent research has employed the latest transformer design for picture captioning, introducing a groundbreaking non-convolutional architecture with a Vision Transformer as the encoder and a generic Transformer as the decoder [25]. Dual-graph convolutional networks with transformers and curriculum learning are explored to investigate the contextual significance of adjacent pictures in image captioning. Employs a transformer-based linguistic decoder to generate captions, while separate GCNs encode the entire image and its objects [26]. ReFormer transformer is proposed to produce features that accurately capture pairwise relationships between objects in photos and relational information [27].

Graphs are used in image captioning research to depict spatial and semantic relationships; two methods include extracting scene graphs from images or textual data. Scene graph models integrate objects and their relationships, employing CNN for object attribute modelling and a dictionary approach, with image descriptions generated via Faster-RCNN, yet neglecting contextual relationships among dataset descriptions [28]. Abstract View Graph (ASG) structure is proposed to capture user intent with greater precision and detail. Which effectively manages both sequence modelling tasks [29].

Pointing technique enhances automated captioning by combining a target-detection model with an LSTM to predict outcomes, addressing issues of overfitting and hallucinations in image captioning. Prior methods lack discrimination, describing image contents universally and failing to capture each image’s unique essence [30]. Previous methods accurately depict image contents but lack individual discrimination, failing to capture each image’s uniqueness.

Image captioning methods are biased toward language policy, neglect visual policy, and hinder the capture of visual context, especially in more extended sequences such as paragraphs. An innovative framework for fine-grained image-to-language generation addresses this gap, providing captioning at the sentence and paragraph levels. Context-Aware Visual Policy (CAVP) framework enhances compositional visual reasoning by incorporating visual context, improving captioning quality through contextual cues, and enriching the user experience in image captioning [31].

Image description methods struggle to encapsulate complex textual and visual details within a single caption, prompting the introduction of Anchor-Captioner to address this limitation by generating multiple captions from varied perspectives and integrating additional scene information, thereby enhancing overall caption quality and comprehensiveness [32]. Researchers introduced a POS guidance module to enhance the model’s picture captioning by determining the significance of image features and word sequences, integrating POS characteristics with the Bi-LSTM model’s output to produce captions with diverse expressions and adherence to grammatical rules, thereby improving accuracy and contextual relevance [33].

**Problem Statement:** The existing descriptions created during testing are typically remarkably similar to those seen during training because models usually use direct representations of the descriptions they encounter. It makes it challenging to achieve human-level performance, leading to numerous repeats and limiting the diversity of generated descriptions. Also, the caption size cannot be varied based on the image contents or user interest. Furthermore, the image context is not taken into account when generating image captions. The objects detected within an image do not relate to each other, and therefore, the existing approaches fail to perceive the actual context within an image’s boundary. So, it is necessary to develop a model capable of extracting semantic information from images and generating meaningful interpretations beyond what is present in the training dataset. The training should focus on removing the underlying content, such as the various senses, or establishing relationships between objects.

In this paper, we have proposed a pipeline describing the comprehensive content of the image. We use a language-generative transformer-based decoder to generate captions of images. It improves the quality of generated captions by capturing both semantic relationships and contextual information between objects in an image. We tested our model on the Flicker8k [9] and MSCOCO datasets [34]. We conducted an expert survey to evaluate the model’s performance. The objectives of this paper are:

1. To evaluate the effectiveness of a generative transformer in producing image captions.
2. To describe the images by contextualizing the object concerning other objects in an image.
3. To integrate positional and object embeddings for improved contextual captioning.

The main contributions of this research are as follows:

1. We propose a context-aware captioning framework that incorporates scene graphs to represent spatial and semantic relationships between objects, enabling more meaningful and structured descriptions rather than simple object enumeration.
2. A generative captioning approach capable of producing original descriptions instead of relying on predefined or template-based sentences, with the additional flexibility to generate captions at varying levels of detail depending on the intended use.
3. An expert-based evaluation demonstrating that integrating relational reasoning and flexible caption generation improves the relevance, accuracy, and descriptive richness of the produced captions compared to conventional approaches.

The rest of this paper is organized as follows: The Materials and Methods section describes the proposed model and the experimental setup used to evaluate it. These are then followed by the Results and Discussion section, which explains the model’s experimental results, the expert survey, and examples of generated captions. The Section Conclusion concludes the paper and represents the expected direction.

## Materials and methods

**Fig 1:**
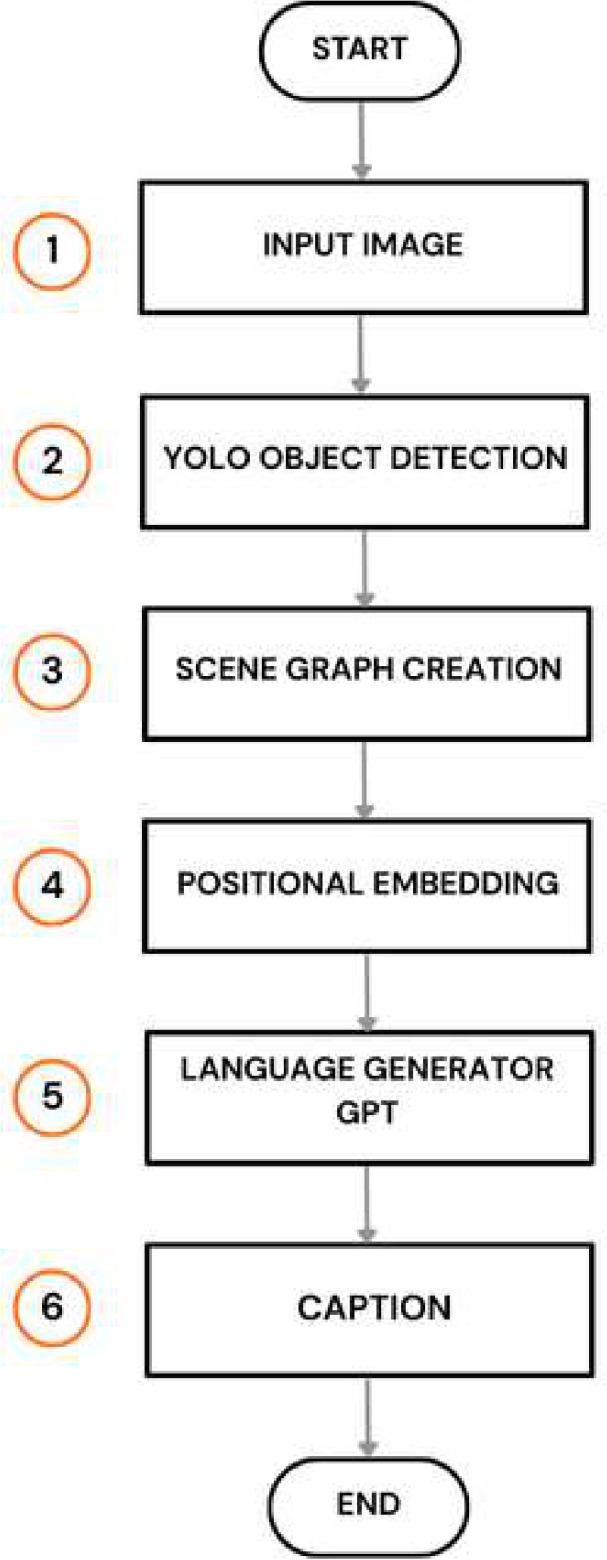
Sequential processing pipeline of the image captioning system. This figure summarizes the main workflow of the methodology from input to caption generation.

**Fig 2:**
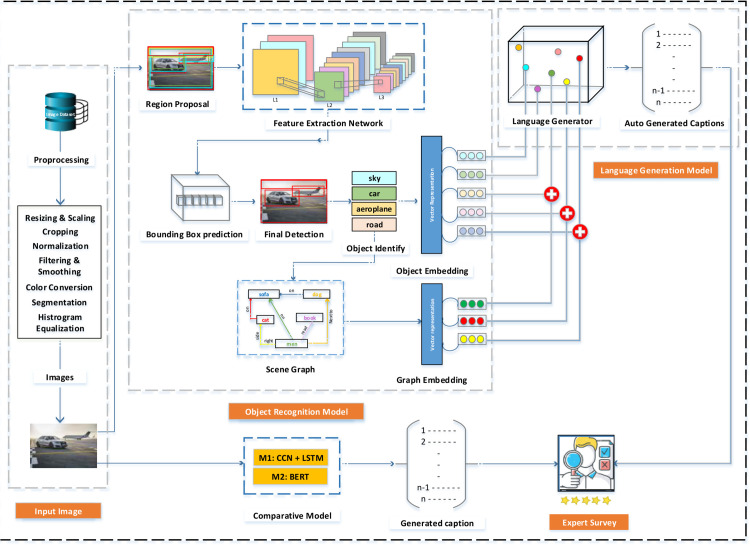
Proposed architecture diagram for automatic caption generation from images using language generation models. The figure illustrates the overall process of the proposed method. It begins with acquiring the dataset and preparing the images through preprocessing. After that, processed images are passed through an encoder to extract meaningful features, which are enriched with positional embeddings to preserve spatial relationships. Finally, these representations are provided to a generative model that produces contextual image captions.

The proposed technique was explained step-by-step, with each methodology step thoroughly discussed, providing a detailed overview of the entire process. The participant featured in the images has provided written informed consent for the publication of these identifiable images, as per the PLOS consent guidelines. Fig 1 represents the sequential steps of the proposed methodology, from input image to generated caption. while Fig 2 shows the internal structure and modular connections of the proposed technique. In our scenario, caption generation is the assigned task. Multiple models are integrated with the state-of-the-art language generator transformer Algorithm 1 outlines the steps of the proposed method in pseudocode. It provides a clear representation of the system’s workflow, ensuring both clarity and reproducibility for implementation.

**Algorithm 1 Proposed Image Caption Generation Framework**

1:  **Input:** Image *I*

2:  **Output:** Caption *C*

3:  $I_{norm}\leftarrow Normalize(Resize(I))$

4:  $R\leftarrow RegionProposal(I_{norm})$

5:  $\left\{B,Labels\}\leftarrow YOLOv4\_Detect(I_{norm}\right)$

6:  G←InitializeGraph()

7:  **for** each object *o* in Labels **do**

8:   AddNode(*G*, *o*)

9:   AssignAttributes(*G*, *o*.*location*, *o*.*size*, *o*.*category*)

10:  **end for**

11:  DefineRelationships(*G*)

12:  **for** each node *v* in *G* **do**

13:   v←PositionEmbedding(v)

14:  **end for**

15:  C←GPT(G)

16:  **return** *C*

### Image preprocessing

**Fig 3:**
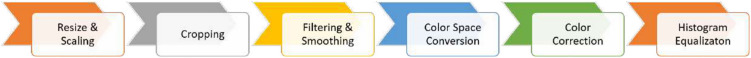
Image pre-processing steps of the proposed methodology. This figure shows the stages of the pre-processing, before training the captioning model.

Image preprocessing was a fundamental step in computer vision, involving manipulating raw images to improve their quality and prepare them for further analysis. The goal of preprocessing was to enhance specific visual features crucial for subsequent processing and analysis, or to reduce undesirable distortions, thereby improving the image data. The efficiency and resilience of computer vision algorithms could be significantly enhanced by optimising input data. Fig 3 illustrates some common preprocessing steps in image analysis. Raw images were first normalised and resized to a fixed resolution, ensuring consistency across datasets. Preprocessing enhanced discriminative features while minimising distortions, thereby improving the robustness of downstream detection and captioning tasks. Formally, image normalisation Eq. (1) ensures uniform pixel distribution across datasets:


$$\begin{array}{c} I^{\prime }=\frac{I-\mu }{\sigma } \end{array}$$ (1)

where μ and σ represent the mean and standard deviation of the dataset.

### Region proposal

**Fig 4:**
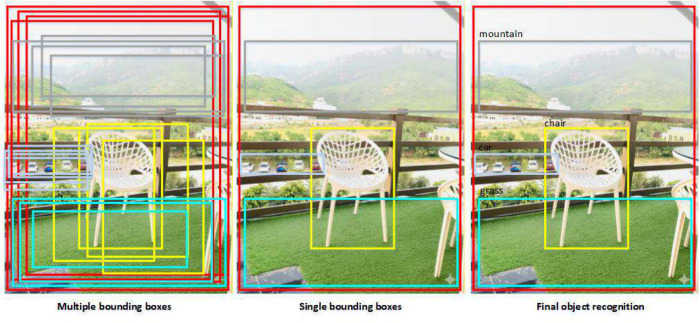
Result of object detection in image based on the region of interest. The figure displays the outcome of object detection, with bounding boxes marking the regions of interest in the image to indicate where objects have been successfully identified.

In image analysis, the Region of Interest (ROI) was the specific area within an image selected for detailed analysis, focusing computational resources on relevant features for tasks like object detection or tracking. Fig 4 illustrates object detection utilising the ROI.

### Feature extraction

Feature extraction was the process of extracting relevant and distinctive information, known as features, from images. In computer vision or image processing, feature extraction involves identifying and capturing meaningful patterns, structures, or characteristics from images or image regions. Feature extraction aims to transform raw data into a more compact, representative representation, retaining important information for subsequent analysis or recognition tasks. These extracted features acted as distinctive descriptors that captured the key characteristics of the data, enabling efficient and effective processing. In computer vision, for each region of interest, a convolutional CSP backbone in YOLOv5 extracts hierarchical features. Feature maps $F_{i,j}$ were computed using standard convolution Eq. (2), where *K* is the convolution kernel, and *I* is the input region:


$$\begin{array}{c} F_{i,j}=\sum_{m,n}I_{i+m,j+n}\cdot K_{m,n} \end{array}$$ (2)

Pooling layers further reduce spatial dimensions while retaining salient information. These features provide a compact yet informative representation for object classification and localization.

### Bounding boxes predictions

A bounding box was a rectangle that surrounded an object and indicated its position. Each bounding box was predicted based on the features extracted from the entire image. We relied on the widely used detection backbone YOLOv5 [35] to extract object-level features. Later versions of the same model could have served this purpose just as well without changing the framework. YOLO predicted bounding boxes along with object class probabilities. Multiple boxes were generated per object, which were then refined using Non-Maximum Suppression (NMS). Fig 4 showed objects detected within bounding boxes. The overlap between a predicted bounding box $B_{p}$ and a ground truth box $B_{gt}$ is quantified via Intersection-over-Union (IoU): Eq. (3) quantifies the overlap between a predicted bounding box $B_{p}$ and a ground truth box $B_{gt}$ using the Intersection-over-Union (IoU) metric:


$$\begin{array}{c} IoU=\frac{\left|B_{p}\cap B_{gt}\right|}{\left|B_{p}\cup B_{gt}\right|} \end{array}$$ (3)

### Scene graph and position embedding

**Fig 5:**
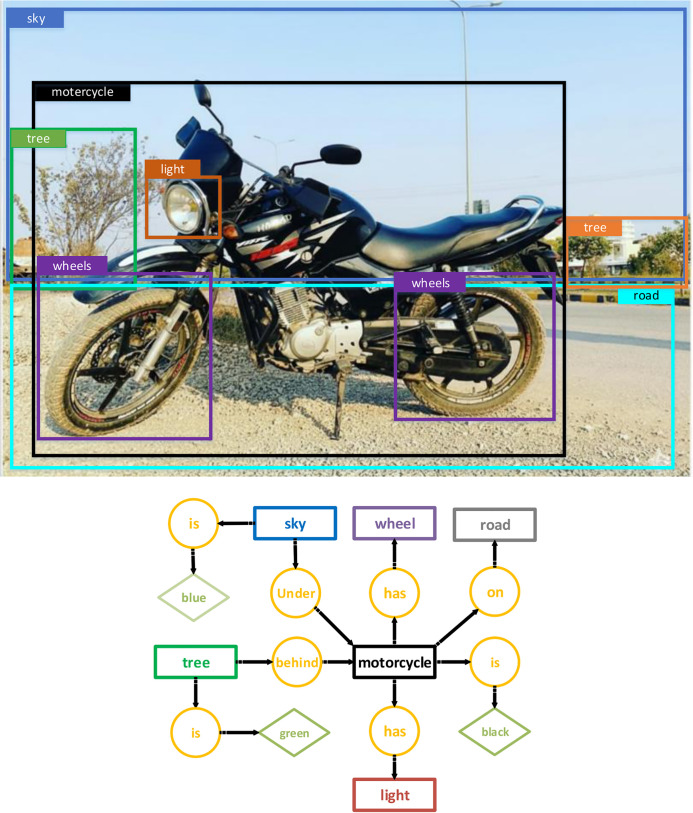
Objects detection and representation of image scene graph. This figure depicts the constructed scene graph, where objects are represented as nodes and their semantic relationships are expressed through edges, offering a structured interpretation of the visual content.

After object detection was completed, a scene graph was constructed to represent the identified objects and define the semantic and spatial relationships among them [36]. These steps allowed the system to transform the detection output into a structured representation rather than treating each detected object individually. Each object served as a node, and relational terms such as “behind,” “under,” “has,” or “on” defined the edges. Fig 5 illustrates the generated scene graph and the relational structure formed based on the detected elements.

Positional embeddings were then applied to encode the spatial arrangement of the objects in the scene graph. It’s necessary because, without positional information, the input to the language model would have remained an unordered list of labels, which typically leads to fragmented or list-style captions. By embedding spatial context, the model received information about how objects were arranged and interacted within the scene. It enabled the captioning model to interpret the input as a coherent structure, resulting in more accurate, context-aware, and logically connected image descriptions. Thus, positional embedding contributed significantly to improving caption coherence by ensuring that the generated sentences reflected both object identity and their spatial relationships.

### Automatic caption generation

After applying position embedding to the input data, the Generative Pre-trained Transformer (GPT-4) [37] processed these tokens enriched with positional context. The model converted these tokens into a vector representation, capturing the image’s semantic content. Using this representation, GPT-4 generated a descriptive image caption that was contextually relevant and reflective of the spatial relationships between objects. The model output filtering step ensured that the generated caption was not only pertinent but also accurate and coherent, making it suitable for describing complex visual scenes.

### Experimental setup

We have performed experiments (code available at GitHub repository) to understand the effectiveness of our approach compared to the methods already proposed. The following sub-sections illustrate the datasets used in the experiments, object detection, scene graph generation, positional embeddings, and final caption generation using the generative model.

All experiments were conducted on the Google Colab platform using Python. The system utilized cloud-based resources, including a GPU and dynamically allocated memory, to support the experimentation. Table 1 presents the complete hardware and system configuration used during the process.

**Table 1:** Hardware and system configuration used in the experiment.

Components	Specification
GPU Type	NVIDIA Tesla T4
GPU Memory	16 GB
CPU Type	Intel Xeon @ 2.20 GHz
RAM	12–25 GB (dynamically allocated)
Average Runtime	20–30 minutes per evaluation cycle

The choice of an appropriate dataset is paramount for task success; considering image captioning, MSCOCO [34] and Flickr8k [9] were relevant options. MSCOCO, known for extensive annotations and diverse tasks, provides a large-scale dataset, while Flickr8k focuses specifically on image captioning, offering a curated collection with captions. MSCOCO’s advantage lies in its versatility, covering various computer vision tasks, but it may be overwhelming for specific applications. Flickr8k excels in its specialisation for image captioning, yet its scale is more limited. Table 2 displays the characteristics of the dataset. Both datasets have significantly contributed to advancing computer vision algorithms and are widely utilised in academic and industrial settings. To conduct performance testing of different models, we have selected 250 images from each dataset and stored them in an “images-set” folder.

**Table 2:** List of selected image datasets for proposed methodology.

#	Dataset Name	No. of Images	No. of Captions	Diskspace	Classes
1	MSCOCO [34]	330k	5 captions per image	13GB	80 Classes
2	Fliker8k [9]	8000	5 captions per image	1GB	Not categorized

The images were preprocessed by resizing them to 416x416 pixels, normalizing the pixel values to a [0, 1] range, and converting them into a blob suitable for input into the YOLOv5 model. The pre-trained YOLOv5 model was used for detecting objects in the image. The model operated strictly in inference mode, and no layers were fine-tuned or retrained. Classifier layers were removed to reduce computational overhead while maintaining detection capability. Non-maximum suppression was applied to eliminate overlapping bounding boxes and retain the most confident predictions. Detected object labels and spatial information were transformed into scene-graph representations. Positional embeddings encoded spatial and semantic relationships among objects, including directional context and relevant object attributes.

Caption generation was performed using the GPT-4 model through the OpenAI API. Structured prompts were constructed using detected objects, positional embeddings, and relational descriptors derived from the generated scene graph. Fixed parameter settings were used throughout the experiments to ensure output consistency. The configuration settings applied during text generation are presented in Table 3. Using these settings, GPT-4 generated captions conditioned on object relationships rather than image pixels. The generated captions reflected contextual meaning grounded in scene structure, and outputs were produced for all images in both datasets.

**Table 3:** Configuration parameters of language gernerative model.

Parameter	Suggested value
Prompt Length (input)	150 tokens
Max Tokens (output)	100 tokens
Temperature	0.7
Top-p	1

### Human subjects and ethics

Ethical approval was waived as no personal data were collected; informed consent was obtained from all participants.

## Results and discussion

Two state-of-the-art image captioning approaches were employed to compare with the proposed model. The initial approach used a combination of CNN and Long Short-Term Memory (LSTM) [38], referred to as the M1 model, whereas the second approach applied the BERT method [39], termed the M2 model. A comparison of these methods with the proposed model, referred to as the M3 model, demonstrated its superior effectiveness in generating precise image captions, highlighting its advancements over existing techniques.

**Fig 6:**
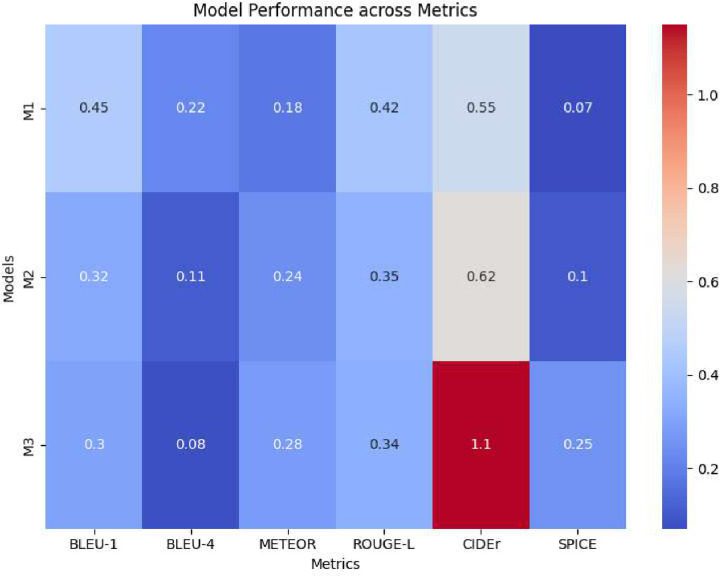
Performance of the proposed model and baseline model across various metrics. This figure presents the evaluation of the model’s performance across various standard metrics.

The performance of the three captioning models (M1, M2, and M3) was compared across BLEU-1/4 [40], ROUGE-L [41], METEOR [42], CIDEr [43], and SPICE [44] using a 500-image test set drawn from MSCOCO and Flickr8k, as shown in Fig 6. Warmer colors represented higher scores. M1 showed reasonable word-level overlap with BLEU-1 (0.45) and ROUGE-L (0.42), but weak semantic understanding, as reflected in its low METEOR (0.18) and SPICE (0.07). M2 improved slightly in semantic and lexical quality, with higher METEOR (0.24) and CIDEr (0.62), though its low BLEU scores indicated difficulty in maintaining consistent n-gram structure.

The proposed model, M3, displayed the strongest semantic and human-aligned performance, achieving the highest CIDEr (1.10) and SPICE (0.25) scores, indicating that it captured object relationships and scene meaning more effectively. Its lower BLEU values were expected for GPT-based generation, as the model paraphrased rather than matching reference captions verbatim. Overall, Fig 6 indicated that M3 produced captions that were more contextually accurate and semantically rich than those from the baseline models.

Standard captioning metrics faced several challenges. They mainly focused on surface-level word matching, but they failed to understand the deeper meaning of the captions. Because of this, they sometimes penalized captions that used different but still correct phrasing. These metrics also didn’t do a good job of evaluating the context, object relationships, or understanding of the scene. They also failed to assess how natural and fluent the language was, missing the human-like quality. Additionally, these metrics didn’t fully show how well a caption represented the actual visual content, and they didn’t consider that there could be multiple correct captions for the same image. That is why expert surveys are used.

The survey was designed to assess caption relevance based solely on textual descriptions, without visualizing the images. A total of 10 experts, holding either BS or MS qualifications across diverse academic disciplines, participated in the evaluation process. We obtained informed consent from all the experts for participating in the survey. No personal data was collected during the study. A dataset comprising 500 images was used for evaluation, with each image containing three captions generated by models M1, M2, and M3, yielding a total of (1–500) captions. To prevent redundancy and capture a wide range of viewpoints, each expert was assigned a minimum of 150 and a maximum of 200 images to evaluate. This distribution strategy helped prevent redundancy and ensured that multiple expert perspectives were represented in the rating process.

Experts evaluated captions from three models (M1–M3) on a five-point relevance scale using Google Forms (available on GitHub repository). Reviewers assessed clarity, completeness, relevance, grammar, conciseness, and audience appropriateness. A pilot test ensured usability, and respondents were given 20 days to complete the evaluation.

To measure the consistency of expert ratings, an inter-coder agreement analysis was conducted. Since more than two raters were involved and rating assignments were distributed, Fleiss’ Kappa (k) was used as the reliability measure. The computed value of k = 0.81 indicated strong agreement among reviewers, suggesting that the ratings were systematic and unlikely to have occurred by chance. This level of agreement strengthened the credibility of the evaluation and supported the validity of the final results.

**Fig 7:**
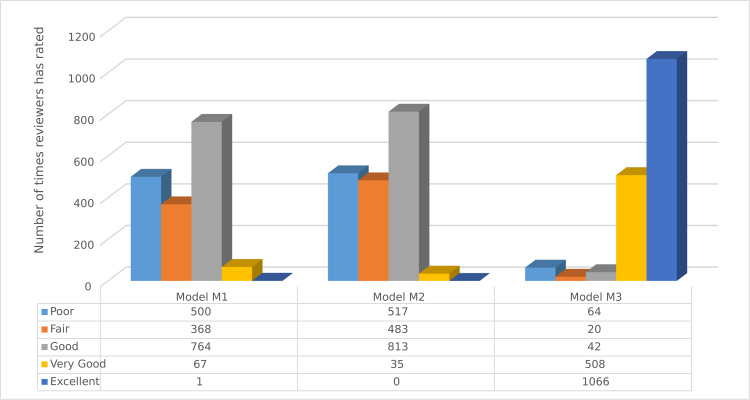
Reviewer evaluations of the generated captions. This figure summarizes the evaluations from ten reviewers, where the scores were based on the completeness of the captions, their semantic quality, and their relevance to the corresponding images.

Fig 7 presents the rating distribution across the three models. M1 and M2 received predominantly fair-to-good scores, with occasional poor evaluations, whereas M3 was rated mainly as very good or excellent, demonstrating consistently higher caption quality. Expert-based relevance outcomes indicate that M3 achieved a significantly higher relevance score (93%), outperforming both M1 (2%) and M2 (5%). It confirms that the proposed model produced the most accurate, descriptive, and context-aware captions.

**Fig 8:**
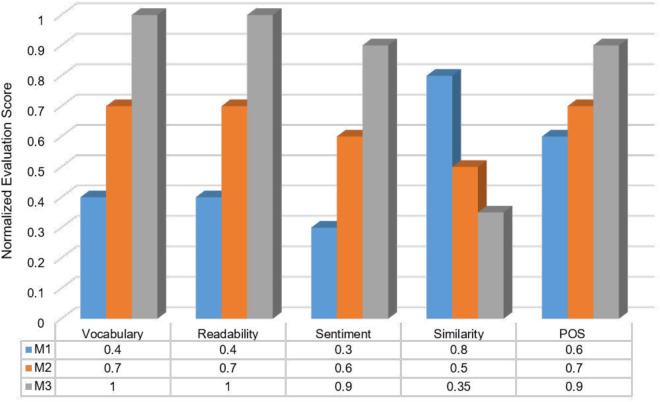
Exploratory Data Analysis of Caption Generation Quality. This figure shows the results of Exploratory Data Analysis (EDA) on captions generated by models M1, M2, and M3. The bar graphs compare the models based on key metrics such as clarity, relevance, and semantic accuracy.

The generated captions were further analyzed to assess how well three distinct models (M1, M2, and M3) performed, examining key aspects such as vocabulary richness, readability (average words per caption), sentiment alignment, similarity to ground-truth captions, and POS diversity. Fig 8 presents a comparative visualization of the normalized evaluation scores (0–1 scale) for these metrics. M1 showed limited vocabulary, lower readability, and reduced structural diversity, reflecting a tendency toward repetitive, memorized phrasing. Its relatively higher similarity score mainly resulted from overlap with training captions rather than meaningful generalization.

M2 demonstrated moderate performance, generating more coherent captions than M1 but still lacking consistency in semantic quality and linguistic variety. M3 consistently produced more descriptive captions with richer vocabulary, stronger readability, and greater linguistic diversity. Although its similarity score was lower, this outcome was expected, as the model focused on semantic accuracy rather than matching reference wording. The exploratory data analysis highlighted the superior linguistic quality and contextual depth achieved by M3.

We further compared the computational efficiency of the proposed pipeline with that of the two baseline models. The CNN–LSTM model was the fastest, requiring 0.42 seconds per image, which resulted in approximately 210 seconds for 500 images. The BERT model, although offering more detailed captions, took 0.67 seconds per image and therefore a total of 336 seconds for 500 images. The YOLOv5 and GPT-4 pipeline required 0.90 seconds per image, with 0.29 seconds for object detection by YOLOv5 and 0.61 seconds for GPT-4 to generate each image caption, thereby amounting to 450 seconds for 500 images. Although our model took longer compared to the baseline models, the quality of the captions was superior, and deeper contextual understanding was possible, thus justifying the increase in the processing time.

Speaking of computational costs, there was no additional financial cost involved due to the runtime of the CNN–LSTM and ViT–BERT models, which were performed on local resources. On the other hand, the usage of GPT-4 did incur financial costs. Assuming 150 input tokens and 100 output tokens per image, approximately $0.015 per image was incurred. Therefore, a total of $7.50 was the cost of processing 500 images solely using GPT-4, a cost not relevant to the comparative baseline models.

The use of GPT-4 for caption generation introduces a computational cost of $0.0075 per image; it is within reasonable bounds in our experiments but becomes prohibitive at larger scales or in real-time applications. For the baseline models, this cost factor does not come into play at all, as no external API is used.

While GPT-4 is really proficient in generating high-quality captions, at the same time, GPT-4 inherits some biases from the dataset on which it has been trained, and these can sometimes manifest as linguistic or even cultural biases, especially when the images themselves depict particular cultural contexts or people. Although these biases can be mitigated to some extent through careful prompt design, the challenge of fully addressing them remains.

The proposed methodology has been evaluated on general-purpose datasets such as MSCOCO and Flickr8k. Still, how well it performs in highly specialized or domain-specific contexts, e.g., medical images, remains unexplored. Despite this, the pipeline’s strong generalization capability would still make it effective in many real-world scenarios. Further work could involve expanding the model’s evaluation to more diverse datasets, though given the model’s robust design across a wide range of image types, we expect these results to remain strong.

## Conclusion

This work introduces a context-aware image captioning pipeline that integrates object detection, scene graphs, positional embeddings, and a generative transformer to produce semantically coherent image descriptions. The proposed YOLOv5–GPT4 pipeline achieved 93% accuracy in expert evaluations, generating more contextually coherent captions than CNN–LSTM and ViT–BERT baselines. By explicitly modeling spatial and relational information, the proposed approach demonstrates strong alignment with human judgment and captures visual context more effectively than traditional methods. Although effective overall, the pipeline remains sensitive to images with sparse or ambiguous visual information, such as minimalistic or partially occluded scenes. Future work will focus on refining the network structure to improve recognition in these scenarios. Furthermore, integrating few-shot techniques could further enhance the quality of generated captions.
